# Sepsis-Induced Myocardial Dysfunction and Its Prognostic Implications for Mortality in the Intensive Care Unit: A Systematic Review

**DOI:** 10.7759/cureus.96887

**Published:** 2025-11-15

**Authors:** Abubakar Gapizov, Ahmad Mohammad, Ahmad Sadiq, Bhavna Singla, Shivam Singla, Simran Kumari, Sunita Kumawat, Asim Iqbal

**Affiliations:** 1 Internal Medicine, NewYork-Presbyterian Brooklyn Methodist Hospital, New York City, USA; 2 Internal Medicine, Hurley Medical Center, Flint, USA; 3 Internal Medicine, Lahore General Hospital, Lahore, PAK; 4 Internal Medicine, Erie County Medical Center Health Campus, Buffalo, USA; 5 Internal Medicine, TidalHealth Peninsula Regional, Salisbury, USA; 6 Internal Medicine, Chandka Medical College, Larkana, PAK; 7 Internal Medicine, St. Francis Medical Center, Lynwood, USA; 8 Internal Medicine, Lahore Medical and Dental College, Lahore, PAK

**Keywords:** biomarkers, critical care outcomes, echocardiography, intensive care, mortality, myocardial dysfunction, sepsis, septic shock

## Abstract

Sepsis-induced myocardial dysfunction (SIMD) is increasingly recognized as a critical determinant of morbidity in intensive care unit (ICU) patients, yet its direct impact on mortality remains uncertain. This systematic review synthesized evidence from randomized controlled trials and observational studies investigating the association between SIMD and outcomes in septic patients. Echocardiographic and biomarker-based assessments consistently demonstrated that SIMD is a frequent and often reversible complication of sepsis, associated with greater disease severity and impaired hemodynamics. Pharmacological interventions such as levosimendan and Xinmailong infusion were found to improve surrogate measures of cardiac function, yet none conferred a significant survival benefit at 28 days. While advanced imaging modalities like speckle-tracking echocardiography enhanced diagnostic sensitivity, heterogeneity in study design, patient populations, and outcome definitions limited the strength of pooled conclusions. Overall, SIMD appears to be a clinically relevant marker of illness severity, but current evidence does not establish it as an independent predictor of mortality. Larger multicenter trials with standardized diagnostic frameworks and long-term follow-up are required to clarify its prognostic role and to determine whether targeted therapies can alter patient outcomes.

## Introduction and background

Sepsis remains a leading cause of morbidity and mortality worldwide, representing a major burden in intensive care units (ICUs). It is characterized by a dysregulated host response to infection that leads to life-threatening organ dysfunction [1]. Despite advances in early recognition, antimicrobial therapy, and supportive care, sepsis continues to account for significant in-hospital deaths, particularly in critically ill patients. Among the complications of sepsis, cardiovascular involvement plays a central role in determining patient outcomes [2].

One of the most recognized cardiovascular manifestations of sepsis is sepsis-induced myocardial dysfunction (SIMD), a reversible impairment of cardiac contractility and relaxation that arises in the setting of systemic inflammation, circulatory dysregulation, and cellular metabolic derangements [3,4]. SIMD has been described in both left and right ventricular dysfunction and is associated with hemodynamic instability, increased vasopressor requirements, and worse clinical outcomes. Its prevalence varies widely, reported in up to 40%-60% of patients with septic shock, depending on diagnostic modalities and definitions used [5].

The pathophysiology of SIMD is complex and multifactorial, involving inflammatory cytokine release, mitochondrial dysfunction, microvascular alterations, and calcium handling abnormalities [6]. Clinically, it can be assessed through echocardiography, hemodynamic monitoring, and emerging biomarkers such as natriuretic peptides and cardiac troponins. While SIMD is often transient and reversible within seven to 10 days in survivors, persistent dysfunction has been associated with increased mortality, prolonged ICU stay, and greater need for circulatory support [7,8]. These temporal dynamics suggest that the prognostic significance of SIMD may depend on both its presence and resolution over time.

Despite growing recognition, the relationship between SIMD and mortality in ICU patients remains inconsistently reported across clinical studies. Some investigations have demonstrated a strong association between SIMD and adverse outcomes, whereas others have found no independent predictive value after adjusting for illness severity and comorbidities. Such discrepancies may reflect heterogeneity in diagnostic criteria, timing of assessment, and patient populations studied.

This systematic review aims to synthesize and critically evaluate the available evidence on the association between sepsis-induced myocardial dysfunction and mortality in ICU patients. By consolidating findings from clinical trials and observational studies, the review seeks to determine whether SIMD independently predicts mortality, characterize its reversibility and time course among survivors, and highlight research priorities for improving diagnostic precision and prognostic stratification in septic patients.

## Review

Materials and methods

Protocol and Reporting Standards

This systematic review was conducted in accordance with the Preferred Reporting Items for Systematic Reviews and Meta-Analyses (PRISMA) 2020 guidelines [9]. PRISMA is an internationally recognized evidence-based framework designed to improve the clarity, transparency, and reproducibility of systematic reviews by standardizing the reporting of key methodological steps. Adhering to PRISMA ensures that readers and future researchers can critically appraise the search strategy, study selection, data extraction, and synthesis processes. The review protocol was developed a priori, outlining objectives, inclusion criteria, and methodological approach to ensure methodological rigor and minimize bias. The final manuscript adheres to PRISMA recommendations regarding study identification, screening, eligibility assessment, data extraction, risk of bias evaluation, and synthesis of evidence.

Research Question and Population, Intervention, Comparison, and Outcome (PICO) Framework

The research question was formulated using the PICO framework [10] to guide the search and selection strategy. The population (P) comprised adult ICU patients diagnosed with sepsis or septic shock. The intervention (I) included studies reporting SIMD, diagnosed using echocardiography, advanced cardiac imaging, or validated biomarkers. The comparator (C) was either standard care, placebo, or patients without myocardial dysfunction. The primary outcome (O) of interest was mortality, while secondary outcomes included the incidence, reversibility, and time course of SIMD, as well as organ dysfunction scores and hemodynamic parameters.

Search Strategy

A comprehensive search was performed in PubMed, Medical Literature Analysis and Retrieval System Online (MEDLINE), and Excerpta Medica database (Embase) from inception until September 2025. The search combined controlled vocabulary (MeSH terms) and free-text keywords related to sepsis, myocardial dysfunction, cardiac dysfunction, intensive care, and mortality. Boolean operators were applied to maximize sensitivity and specificity, for example: (“sepsis” OR “septic shock”) AND (“myocardial dysfunction” OR “cardiac dysfunction” OR “myocardial depression”) AND (“mortality” OR “outcome” OR “intensive care”). Reference lists of eligible articles and relevant reviews were manually screened to capture additional studies. While the search primarily targeted English-language publications, one non-English study meeting all inclusion criteria was incorporated after translation and data verification.

Eligibility Criteria

Studies were considered eligible if they met the following criteria: prospective or retrospective randomized controlled trials, case-control, or cohort studies that included adult ICU patients diagnosed with sepsis or septic shock according to established clinical definitions. Eligible studies were required to report sepsis-induced myocardial dysfunction, identified through echocardiography (including strain imaging), advanced cardiac imaging, or validated cardiac biomarkers. Mortality had to be reported as a primary or secondary outcome, with additional data on reversibility or hemodynamic recovery included when available. Studies limited to pediatric or animal models, narrative reviews, case reports, or conference abstracts were excluded.

Study Selection

Two independent reviewers screened all titles and abstracts retrieved from the search. Full-text review was then performed to confirm eligibility. Discrepancies were resolved by consensus or consultation with a third reviewer. A PRISMA flow diagram was constructed to illustrate the process of identification, screening, eligibility, and inclusion.

Data Extraction

Data were extracted using a standardized form developed for this review. Extracted variables included study characteristics (author, year, country, and design), population demographics, diagnostic methods for myocardial dysfunction, comparator details, primary and secondary outcomes, and mortality data. When available, the duration and reversibility of myocardial dysfunction were also documented. Data extraction was independently performed by two reviewers, with discrepancies reconciled by consensus to minimize bias.

Risk of Bias Assessment

The methodological quality of included studies was assessed using appropriate validated tools. For randomized controlled trials, the Cochrane Risk of Bias 2.0 tool [11] was applied, evaluating domains such as randomization, deviations from intended interventions, missing outcome data, measurement of outcomes, and reporting bias. For observational studies, the Newcastle-Ottawa Scale [12] was employed, focusing on selection, comparability, and outcome assessment. Each study was assigned a qualitative risk of bias judgment (low risk, some concerns, or high risk).

Data Synthesis

Given the clinical and methodological heterogeneity across included studies, a narrative synthesis was performed. Key outcomes were summarized in tabular and descriptive form, with emphasis on the relationship between SIMD and mortality. Quantitative pooling through meta-analysis was not performed due to variability in definitions of myocardial dysfunction, diagnostic modalities, and outcome measures. Instead, results were critically compared to highlight consistencies, contradictions, and gaps in the evidence base.

Results

Study Selection Process

**Figure 1:**
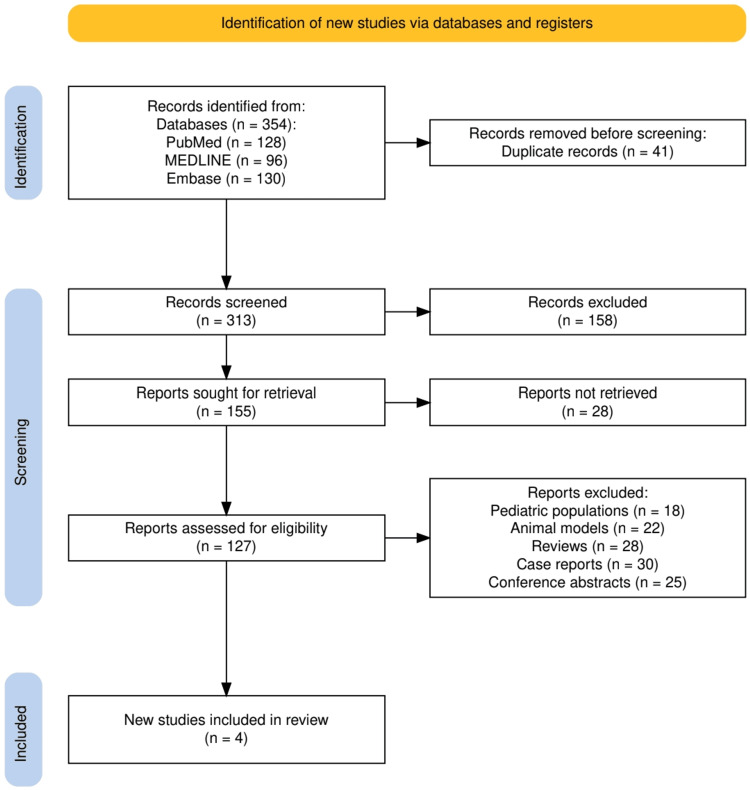
The PRISMA flowchart represents the study selection process. PRISMA: Preferred Reporting Items for Systematic Reviews and Meta-Analyses; MEDLINE: Medical Literature Analysis and Retrieval System Online; Embase: Excerpta Medica database

A total of 354 records were identified through database searching, including 128 from PubMed, 96 from MEDLINE, and 130 from Embase. After the removal of 41 duplicates, 313 studies were screened, of which 158 were excluded at the title and abstract level. Full-text assessment was performed for 127 articles, with 123 subsequently excluded for reasons such as pediatric populations (n = 18), animal models (n = 22), reviews (n = 28), case reports (n = 30), and conference abstracts (n = 25). Ultimately, four studies met the eligibility criteria and were included in this systematic review. The detailed selection process is illustrated in Figure 1.

Characteristics of the Selected Studies

The key characteristics of the studies included in this review are summarized in Table 1. The selected clinical trials and observational studies varied in design, sample size, and diagnostic criteria for SIMD. Randomized controlled trials primarily evaluated pharmacologic interventions such as levosimendan and Xinmailong for their effects on myocardial function, whereas observational and case-control studies focused on the diagnosis, incidence, and reversibility of dysfunction using echocardiographic techniques, including speckle-tracking, and cardiac biomarkers. Across all studies, SIMD was assessed through imaging-based parameters or biochemical markers, with outcomes encompassing the incidence and resolution of dysfunction, hemodynamic alterations, organ failure scores, ICU length of stay, and mortality. Although several studies reported transient improvements in myocardial performance and surrogate measures, none demonstrated a consistent reduction in short-term or 28-day mortality, underscoring the ongoing uncertainty regarding the prognostic significance of SIMD and the effectiveness of targeted therapeutic strategies in critically ill ICU populations.

**Table 1:** Characteristics of included studies evaluating SIMD and mortality outcomes in ICU patients SIMD: sepsis-induced myocardial dysfunction; ICU: intensive care unit; RCT: randomized controlled trial; XMLI: xinmailong infusion; BNP: b-type natriuretic peptide; CTNI: cardiac troponin I; NT-PROBNP: n-terminal pro–b-type natriuretic peptide; SOFA: sequential organ failure assessment; GLS: global longitudinal strain; LVEF: left ventricular ejection fraction; SV: stroke volume Data for Xu et al. (2018) [16] were extracted from a Chinese-language publication following independent translation and verification.

Author (Year)	Study Design	Population (n)	Diagnostic Method for SIMD	Comparator	Primary Outcome(s)	Mortality Findings
He et al. (2021) [13]	RCT, multicenter, double-blind	192 ICU sepsis patients (96 XMLI, 96 placebo)	Echocardiography (diastolic dysfunction on Day 5) + BNP levels	Xinmailong infusion vs placebo	Incidence of SIMD in the ICU	28-day mortality: 36/192 (18.8%), no significant difference (P=0.45)
Antcliffe et al. (2019) [14]	Subgroup analysis of RCT (LeoPARDS trial)	ICU septic shock patients with/without cardiac dysfunction (n=516 overall; subgroup analyzed by biomarkers)	Biochemical evidence: troponin I (cTnI), NT-proBNP	Levosimendan vs placebo (standard care)	SOFA score, 28-day mortality	No mortality benefit with levosimendan; 28-day mortality unchanged
Ng et al. (2016) [15]	Case-control study	33 septic shock patients vs 29 sepsis (no shock) controls	Speckle tracking echocardiography (global longitudinal strain, GLS)	Sepsis without shock	Myocardial strain assessment, reversibility of dysfunction	SIMD associated with worse SOFA and hemodynamics; mortality not directly reported
Xu et al. (2018) [16]	RCT, prospective, controlled	30 elderly septic shock patients with LVEF ≤50% (15 levosimendan, 15 dobutamine)	Echocardiography (LVEF, SV) + lactate	Levosimendan vs dobutamine	Cardiac function (LVEF, SV), lactate, ICU stay, ventilation time	28-day mortality was similar between groups (no significant difference, P>0.05)

Quality Assessment

The quality assessment of the included studies is presented in Table 2. Overall, the multicenter randomized trial demonstrated the most robust methodology, with low risk of bias due to adequate randomization, blinding, and complete outcome reporting. However, subgroup analyses within larger trials carry inherent risks of bias related to non-pre-specified comparisons and potential multiplicity. Observational evidence, while providing valuable mechanistic insights, was limited by small sample sizes, single-center recruitment, and selection bias, despite efforts to use matched controls. Single-center randomized trials also introduced concerns due to incomplete reporting of allocation procedures and blinding. Collectively, while the body of evidence provides important clinical insights into sepsis-induced myocardial dysfunction, the overall quality is mixed, underscoring the need for larger, well-designed multicenter trials with standardized definitions and outcomes.

**Table 2:** Risk of bias assessment of included studies on SIMD RCT: randomized controlled trial; RoB 2.0: Cochrane Risk of Bias 2 tool; NOS: Newcastle–Ottawa Scale; BNP: B-type natriuretic peptide; SIMD: sepsis-induced myocardial dysfunction Data for Xu et al. (2018) [16] were extracted from a Chinese-language publication following independent translation and verification.

Author (Year)	Study Design	Tool Applied	Risk of Bias Assessment
He et al. (2021) [13]	RCT, multicenter, double-blind	Cochrane RoB 2.0	Low risk overall. Adequate randomization and blinding, balanced groups. Mortality data are complete. Some concern: outcome assessment of SIMD (echo, BNP) may have observer bias.
Antcliffe et al. (2019) [14]	Subgroup analysis of RCT (LeoPARDS trial)	Cochrane RoB 2.0 (subgroup analysis)	Some concerns. The main trial had low RoB, but subgroup analyses introduce potential bias (not pre-specified, risk of multiplicity). Mortality outcome robust, but subgroup interaction could be biased.
Ng et al. (2016) [15]	Case-control study	Newcastle–Ottawa Scale (NOS)	Moderate risk. Strength: matched controls for age, sex, risk factors. Weakness: single-center, small sample, possible selection bias. Outcome assessment (strain imaging) is objective, but mortality is not directly reported.
Xu et al. (2018) [16]	RCT, single-center, elderly cohort	Cochrane RoB 2.0	Some concerns. Randomization stated but not fully detailed, small sample size (n=30) → risk of imbalances. Blinding was not clearly described. Outcomes (LVEF, SV, lactate), objective, and mortality reporting are complete.

Discussion

Overview of Findings

This systematic review demonstrates that SIMD is a common and clinically relevant complication among critically ill patients with sepsis and septic shock. Across the included studies, SIMD was identified using echocardiography, advanced imaging techniques such as speckle-tracking, and circulating biomarkers including troponin and NT-proBNP. The reported incidence varied according to diagnostic criteria, with He et al. [13] noting diastolic dysfunction in 42.7% of patients in the Xinmailong group and 63.5% in the placebo group by day 5, underscoring the high prevalence of cardiac involvement in this population. Importantly, several studies also documented reversibility of myocardial impairment among survivors, suggesting that SIMD often represents a transient, adaptive response rather than permanent myocardial injury.

Mortality outcomes, however, were less consistent. Although all trials recognized the prognostic significance of cardiac dysfunction during sepsis, none demonstrated a statistically significant difference in 28-day mortality between intervention and control groups (He et al., 2021 [13]; Xu et al., 2018 [16]; Antcliffe et al., 2019 [14]). These findings imply that while SIMD reflects severe systemic inflammation and multi-organ stress, it may serve more as a marker of overall disease burden rather than an independent determinant of mortality.

Comparison with Previous Literature

Our findings both align with and extend prior literature on SIMD. Earlier observational studies reported mortality rates of 40%-70% in SIMD patients, consistently higher than in those without cardiac involvement, and posited myocardial dysfunction as an independent predictor of death. However, the interventional and ICU-based studies included in this review challenge that interpretation. For example, the LeoPARDS subgroup analysis (Antcliffe et al., 2019 [14]) demonstrated no mortality reduction with levosimendan despite biochemical and echocardiographic evidence of dysfunction, aligning with prior meta-analyses suggesting that inotropes may improve hemodynamics without conferring survival benefit. Similarly, Xu et al. [16] observed improved left ventricular ejection fraction and stroke volume in elderly septic patients treated with levosimendan compared to dobutamine, but without an associated decrease in 28-day mortality. Collectively, these results reinforce that improvement in surrogate cardiac metrics does not necessarily translate into better clinical outcomes, highlighting the complexity of differentiating direct myocardial injury from the broader systemic pathophysiology of sepsis.

Methodological Considerations and Pathophysiological Insights

The studies included in this review varied considerably in design, sample size, and methodological rigor, which directly influences the strength and generalizability of their conclusions. Large multicenter randomized controlled trials, such as He et al. [13], provide relatively robust evidence with lower risk of bias due to randomization and blinding, whereas smaller single-center studies like Xu et al. [16] may be underpowered to detect mortality differences and are more prone to type II error. Observational designs, including Ng et al. [15], contributed valuable diagnostic and pathophysiological insights but are inherently susceptible to confounding, referral, and selection bias. Moreover, the lack of a standardized definition of SIMD remains a major limitation: while some studies defined it by echocardiographic criteria such as diastolic or systolic dysfunction, others relied on biomarkers or speckle-tracking strain analysis. This heterogeneity in case definitions and diagnostic methods complicates data synthesis and likely contributes to the observed variability in reported incidence and outcome associations across studies.

The pathophysiology of SIMD is multifactorial, encompassing inflammatory cytokine cascades, mitochondrial injury, microvascular ischemia, and autonomic dysregulation. This interplay can lead to transient myocardial “stunning” and impaired contractility, which may resolve as the underlying sepsis improves. Indeed, recovery of cardiac function in survivors has been documented within days, as noted by Ng et al. [15], suggesting that SIMD often represents a reversible, adaptive response rather than permanent damage. Clinically, this distinction is critical: therapies that enhance contractility may improve hemodynamic profiles but not necessarily survival, as the primary determinants of outcome in sepsis are systemic inflammation, organ failure, and microcirculatory compromise rather than isolated myocardial impairment [17-19]. Consequently, SIMD should be interpreted as a dynamic indicator of sepsis severity and physiological stress rather than a direct cause of death.

Clinical Implications and Prognostic Value

The evidence reviewed suggests that SIMD has considerable potential as a risk stratification marker in ICU patients with sepsis. Routine echocardiographic assessment, particularly when incorporating advanced modalities such as speckle-tracking, combined with cardiac biomarkers like NT-proBNP and troponin, can yield prognostic insights beyond conventional hemodynamic parameters [20]. Such integration may help identify patients at heightened risk of organ failure or prolonged ICU stay. However, translating these diagnostic findings into therapeutic benefit remains challenging. Pharmacologic interventions, including levosimendan, have shown improvements in myocardial performance indices but have consistently failed to reduce 28-day mortality [21], reinforcing that correction of cardiac dysfunction alone is insufficient to alter survival in sepsis. Consequently, SIMD should be recognized primarily as a marker of disease severity that warrants close monitoring and individualized supportive management rather than as a direct therapeutic target.

Strengths and Limitations

This systematic review has several strengths, including a comprehensive literature search, adherence to PRISMA guidelines, and a focus on clinically relevant outcomes in critically ill populations. Synthesizing data from both interventional and observational studies provides a balanced appraisal of SIMD’s prognostic role in ICU settings. Nonetheless, key limitations must be acknowledged. The number of high-quality randomized controlled trials remains limited, and several included studies are small, single-center investigations with modest sample sizes, reducing statistical power and external validity. Heterogeneity in diagnostic definitions, ranging from biomarker-based to imaging-based criteria, further complicates data comparability. One included study was published in Chinese, and although data were extracted following independent translation and verification, minor interpretation bias cannot be excluded. Finally, potential publication bias remains possible, as studies with neutral or negative findings may be underrepresented.

Future Directions

To strengthen the evidence base, future research should focus on large, multicenter prospective trials employing standardized diagnostic criteria for SIMD. Combining echocardiographic strain imaging with biomarker profiling could help establish a reproducible, clinically actionable definition of myocardial dysfunction. Mechanistic studies are also needed to clarify whether SIMD independently contributes to mortality or merely reflects systemic severity [22,23]. Furthermore, investigation of novel therapeutic approaches beyond traditional inotropes, such as immunomodulatory, mitochondrial, or metabolic agents, may yield more meaningful clinical benefit. Finally, integrating SIMD-based risk stratification into sepsis care bundles could enhance early management strategies, improve triage precision, and ultimately optimize patient outcomes.

## Conclusions

In summary, SIMD is a frequent and clinically significant complication among ICU patients with sepsis and septic shock. Although it is consistently associated with greater disease severity, vasopressor dependence, and organ dysfunction, current evidence does not conclusively establish it as an independent predictor of mortality. This uncertainty largely stems from methodological variability, limited sample sizes, and heterogeneity in diagnostic criteria across studies. Moreover, while SIMD often resolves in survivors within days, its persistence and long-term prognostic implications remain unclear. Present data do not support the use of targeted inotropic therapy such as levosimendan as a mortality-reducing strategy. Future multicenter studies employing standardized definitions, longitudinal follow-up, and mechanistic endpoints are warranted to determine whether directly addressing SIMD can meaningfully improve survival and recovery in this high-risk population.
